# Interfacial Engineering with One-Dimensional Lepidocrocite TiO_2_-Based Nanofilaments for High-Performance Perovskite Solar Cells

**DOI:** 10.1021/acsomega.4c09516

**Published:** 2024-12-13

**Authors:** Shrabani Panigrahi, Hussein O. Badr, Jonas Deuermeier, Santanu Jana, Elvira Fortunato, Rodrigo Martins, Michel W. Barsoum

**Affiliations:** † i3N/CENIMAT, Department of Materials Science, NOVA School of Science and Technology, and CEMOP/UNINOVA, Campus de Caparica, 2829-516 Caparica, Portugal; ‡ Department of Material Science and Engineering, Drexel University, Philadelphia 19104, Pennsylvania, United States

## Abstract

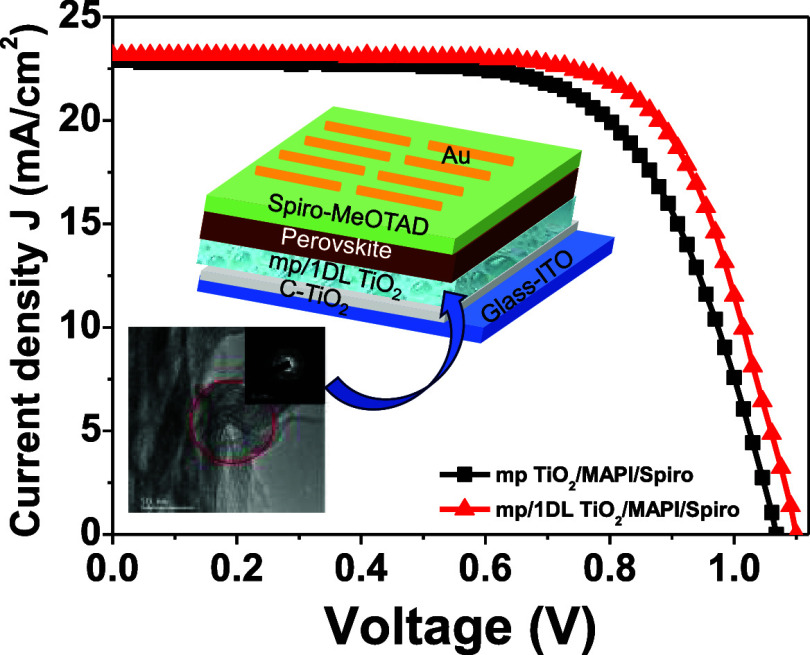

The optimization of nonradiative recombination losses through interface engineering is key to the development of efficient, stable, and hysteresis-free perovskite solar cells (PSCs). In this study, for the first time in solar cell technology, we present a novel approach to interface modification by employing one-dimensional lepidocrocite (henceforth referred to as 1DL) TiO_2_-based nanofilaments, NFs, between the mesoporous TiO_2_ (mp TiO_2_) and halide perovskite film in PSCs to improve both the efficiency and stability of the devices. The 1DLs can be easily produced on the kilogram scale starting with cheap and earth-abundant precursor powders, such as TiC, TiN, TiB_2_, etc., and a common organic base like tetramethylammonium hydroxide. Notably, the 1DL deposition influenced perovskite grain development, resulting in a larger grain size and a more compact perovskite layer. Additionally, it minimized trap centers in the material and reduced charge recombination processes, as confirmed by the photoluminescence analysis. The overall promotion led to an improved power conversion efficiency (PCE) from 13 ± 3.2 to 16 ± 1.8% after interface modification. The champion PCE for the 1DL-containing devices is 17.82%, which is higher than that of 16.17% for the control devices. The passivation effect is further demonstrated by evaluating the stability of PSCs under ambient conditions, wherein the 1DL-containing PSCs maintain ∼87% of their initial efficiency after 120 days. This work provides not only cost-effective, novel, and promising materials for cathode interface engineering but also an effective approach to achieve high-efficiency PSCs with long-term stability devoid of encapsulation.

## Introduction

Extensive research has been devoted to finding fundamental solutions to ever-growing concerns about climate change and energy security. One route is by replacing the current carbon-rich fossil fuels with renewable and environmentally friendly zero-emission energy sources.^1−3^ Among them, photovoltaics (PVs) have gained increasing research interest owing to their low cost, sustainability, and reliability.^4,5^ Despite long-developed PV technologies involving conventional silicon-, organic-, and dye-sensitized solar cells, among others, it is still difficult to meet the market demand for cost-effective, as well as increased efficiency and lifetime PV technologies.

Metal halide perovskite solar cells (PSCs) are regarded as one of the best options that could be commercialized on a large scale because they are both inexpensive and efficient.^6−8^ Since the first report in 2009,^9^ the power conversion efficiency (PCE) of PSCs has rapidly grown to 26% currently.^10^ A number of strategies have been proposed to improve both the stability and efficiency of PSCs. They include advancement in charge-transporting materials,^11,12^ optimized perovskite compositions,^13,14^ and improved deposition techniques.^15,16^

The interface engineering method is also considered as one of the most effective approaches to enhance PSCs' efficiencies and stability.^17−20^ In a typical mesoporous (n–i–p) PSC device, the perovskite absorber layer is positioned between two interfaces with the charge transport layers: one is the electron transport layer (ETL) and the other is the hole transport layer (HTL). These interfaces play a critical role in both determining the properties of the perovskite layers and regulating the solar cell’s overall performance.^21^ By considering the recent progress in interface engineering, the interfacial region between the ETL and perovskite material plays a more crucial role in ensuring the development of highly efficient and stable PSCs. Kumar et al.^17^ employed 8-oxychinoline (8-Oxin) at the mesoporous titanium dioxide (mp TiO_2_)/perovskite interface to tailor the surface of the latter and improve both PV performance and stability. A simple and cheap method was applied by Khaleel and Ahmed^22^ to modify the interface between the ETL and perovskite by wetting the mp TiO_2_ ETL with a mixture of dimethyl sulfoxide (DMSO) and γ-butyrolactone (GBL). Wetting by the ETL changed the perovskite’s grain sizes and passivated grain boundaries, which helped electron extraction at the ETL/perovskite interface, reduced charge accumulation, and increased solar cell performance. Panigrahi et al.^18^ utilized a very thin layer of zinc oxysulfide (ZnOS) between the SnO_2_ ETL and halide perovskite film to passivate the defects and stop the nonradiative recombination losses, which helped to raise the open-circuit voltage (*V*_OC_) and efficiency. Dong et al.^23^ reported that the TiO_*x*_ ETL modifications accomplished with a thin layer of PEO and the devices based on the modified ETL enhanced both *V*_OC_ and short-circuit current density (*J*_SC_), which provided an overall conversion efficiency improvement of about 15% when compared to devices with TiO_*x*_ alone.^24,25^

We recently developed one-dimensional (1D) TiO_2_-based nanofilaments (NFs) by reacting earth-abundant, nontoxic, Ti-containing precursors like TiC, TiB_2_, TiSi_2_, among others, with quaternary ammonium hydroxides, mostly tetramethylammonium hydroxide (TMAH), at ≈80 °C and 1 atm. for tens of hours.^26,27^ The formed nanostructures are 1D lepidocrocite titanate-based NFs henceforth referred to as 1DL.^28,29^ The 1DL cross-section is in the 5 × 8 Å^2^ range, and 1 g of the material spans ≈600 million kilometers. Using a combination of scanning transmission electron microscopy, Raman spectroscopy, and X-ray diffraction (XRD) results, we demonstrated that the NFs comprised 2 layers of Ti atoms arranged in a zigzag structure.^30^ Later, Lagunas et al.^31^ demonstrated that the stacking configuration of Ti layers can be altered via ion exchange with Li, Na, and TMA cations. The 1DLs' extreme dimensions result in a band gap energy, *E*_g_, of 4.1 eV. This record *E*_g_ for TiO_2_-based materials is due to quantum confinement and indirectly confirms our extreme dimensions.^26,32,33^ Said otherwise, our 1DLs are fundamentally different from other TiO_2_-based nanostructures in the literature. They outperform a commercial TiO_2_ nanomaterial (Evonik Aeroxide TiO_2_ P25) in a wide range of applications. In photocatalytic water splitting, our 1DLs showed an apparent quantum yield as high as 11.7%, along with high stability in water for >6 months, 300 h, of which were under 1 sun illumination.^34^ In terms of dye degradation, we showed that certain dyes sensitize our 1DLs, allowing them to degrade dyes in the visible spectrum; P25 was totally inert under the same testing conditions.^35^ In electrocatalytic oxygen evolution reactions, our results showed that doping the 1DLs with Ni and Fe rendered them not only as active as, but also more durable than, Ir black or NiFe layered double hydroxides in alkaline electrolytes.^36^ In water purification, we demonstrated that 1 g of 1DLs is capable of adsorbing up to 424 mg of uranium (U^4+^), rendering water contaminated by this actinide potable.^37^ In energy storage, 1DL electrodes performed well in lithium-ion and lithium–sulfur systems.^26,38^ Lastly, composites of our 1DLs with a repairable, dynamic covalent thiol–yne network resulted in a 500 times increase in the modulus at 60 wt % filler when compared to the pristine polymer.^39^

These positive attributes and properties begged the question: what alterations in cell performance might arise from fabricating the PSCs mentioned here? The PSCs have the following configuration: Indium tin oxide (ITO)/compact TiO_2_ (c-TiO_2_)/mp TiO_2_/1DL/perovskite/HTL/Au. The incorporation of 1DL TiO_2_ for interface engineering in PSCs has markedly improved charge transport and reduced charge recombination, resulting in significant increases in both *V*_OC_ and FF. Moreover, our findings demonstrate that this enhancement boosts solar cell efficiencies by approximately 2% and significantly fortifies their stability.

## Results and Discussion

**Figure 1:**
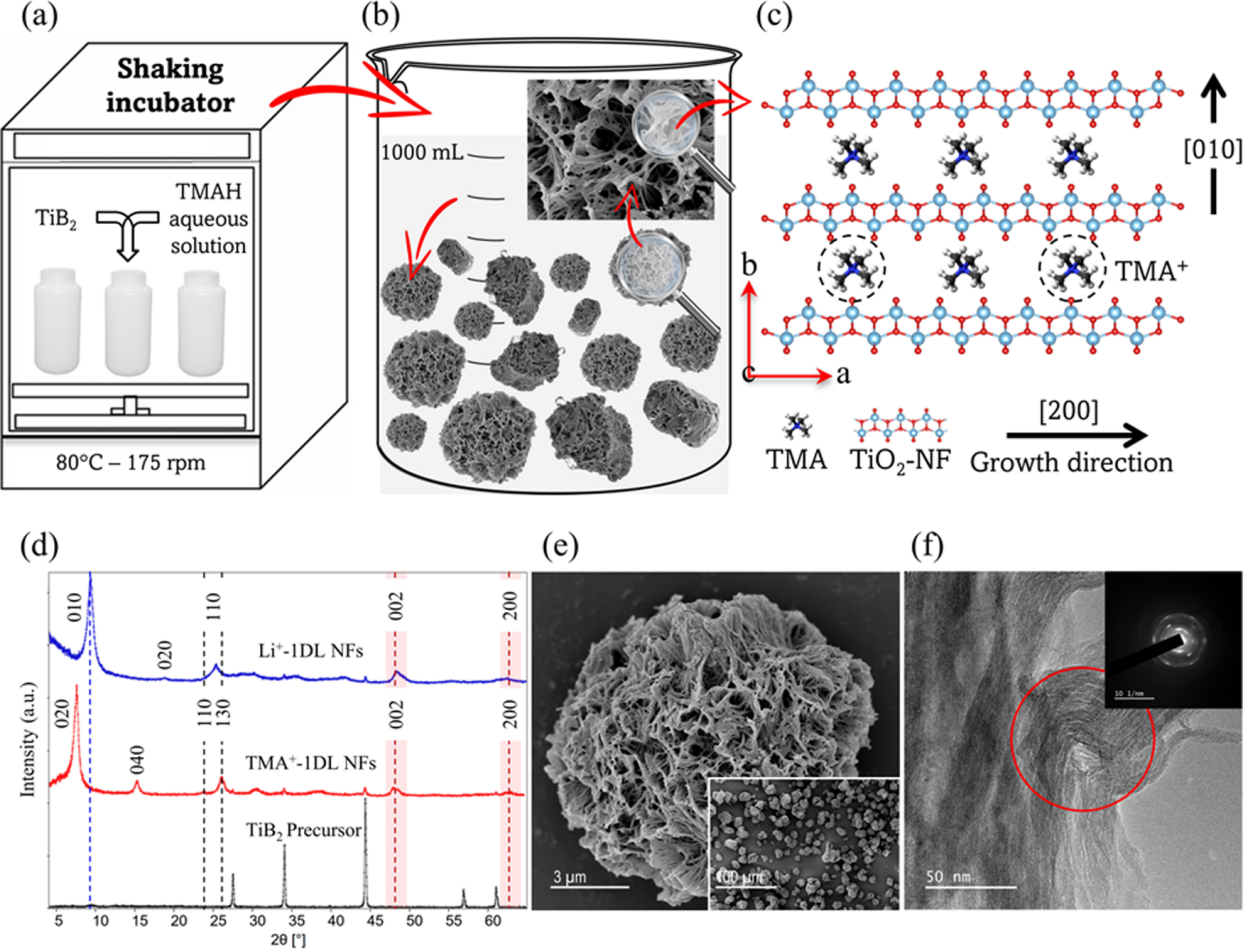
Processing details and characteristics of 1DL powders obtained by heating a TiB_2_ precursor in TMAH solution at 80 °C for 5 days. (a) Schematic showing a temperature-controlled shaker used to convert TiB_2_ powder into 1DL powders and (b) washing protocol followed to remove excess TMAH after the reaction. (c) DFT-generated lepidocrocite titanate structure showing 2 Ti atom-thick ribbons growing and stacking along *a-* and *b*-crystallographic directions, respectively. (d) XRD pattern of TiB_2_ precursor powder (bottom black curve), TiB_2_-derived 1DLs after the reaction and washing with EtOH (middle red curve), and after treatment in a 5 M LiCl solution (top blue curve). Dashed vertical lines denote peak indices. Vertical red bands denote the 002 and 200 nonbasal reflections of the lepidocrocite titanate structure. (e) Typical SEM micrograph of 1DL porous hierarchical particles after LiCl treatment. Inset is low magnification imaging of free-flowing and nonagglomerating 1DL hierarchical particles. (f) TEM micrograph of 1DL bundle oriented along the fiber axis. The inset shows the SAD pattern of area bounded by a red circle and is characterized by two sets of arcs.

Figure 1a sketches the 1DL fabrication process, in which a mixture of 20 g of TiB_2_ precursor powders and 200 mL of TMAH aqueous solution is allowed to react by shaking at 80 °C for 5 days. The resulting slurry is rinsed with 200 proof ethanol, EtOH, 5 times until neutralization (pH ≈ 7) using an overhead mixer (Figure 1b), before allowing it to dry in open air at 50 °C for 24 h. When dehydrated straight from EtOH, the 1DLs self-assemble in porous hierarchical structures, as shown in the schematic in Figure 1b. A full description of the synthesis and characterization of these 1DLs porous hierarchical structures can be found in the Experimental Section below and in recent publications.^29^

The synthesized 1DLs are composed of NFs that are 2 Ti atoms thick and arranged in a zigzag pattern denoting a 1D lepidocrocite titanate structure (see Figure 1c). The red curve in Figure 1d presents the XRD signature of TMA-intercalated 1DLs with a *d*_020_ spacing at 11.5 Å, along with the characteristic nonbasal reflections, viz. 110, 130, 002, and 200 peaks. The NFs grow in the *a*-direction and stack in the *b*-direction.

To rid the interfilamentous space of TMA^+^, we typically wash the powders with a LiCl solution treatment, wherein Li ions replace their TMA counterparts via ion exchange. From the resulting XRD patterns (blue pattern in Figure 1d), the following points are salient: (i) the low angle peak is now shifted to a *d*-spacing of 9.5 Å and (ii) the −ABA– stacking configuration, featured by the 110/130 pair of peaks around 25° 2θ values, is replaced by an −AAA– type, which is recognized by the presence of one broad 110 peak around the same 2θ (blue curve in Figure 1d). In both cases, the 002 and 200 peaks line up, confirming the NF’s lepidocrocite titanate structure. Morphologically, Figure 1e reveals a typical scanning electron microscopy (SEM) micrograph of the resulting 1DL porous hierarchical structure after the LiCl treatment. Noteworthily, the product obtained is a free-flowing, nonagglomerating powder, as shown in the low-magnification SEM image in the inset of Figure 1e. This powder is comprised of sphere-like mesoporous hierarchical particles, as shown in Figure 1e, and will henceforth be referred to as 1DL.

At the transmission electron microscopy (TEM) level, typical nanobundles composed of a multitude of 1DLs are observed (Figure 1f). The corresponding selected area diffraction (SAD) pattern, as shown in the inset of Figure 1f, depicts the two sets of arcs with *d*-spacing values corresponding to the XRD peaks at ∼26° and ∼48° 2θ.

**Figure 2:**
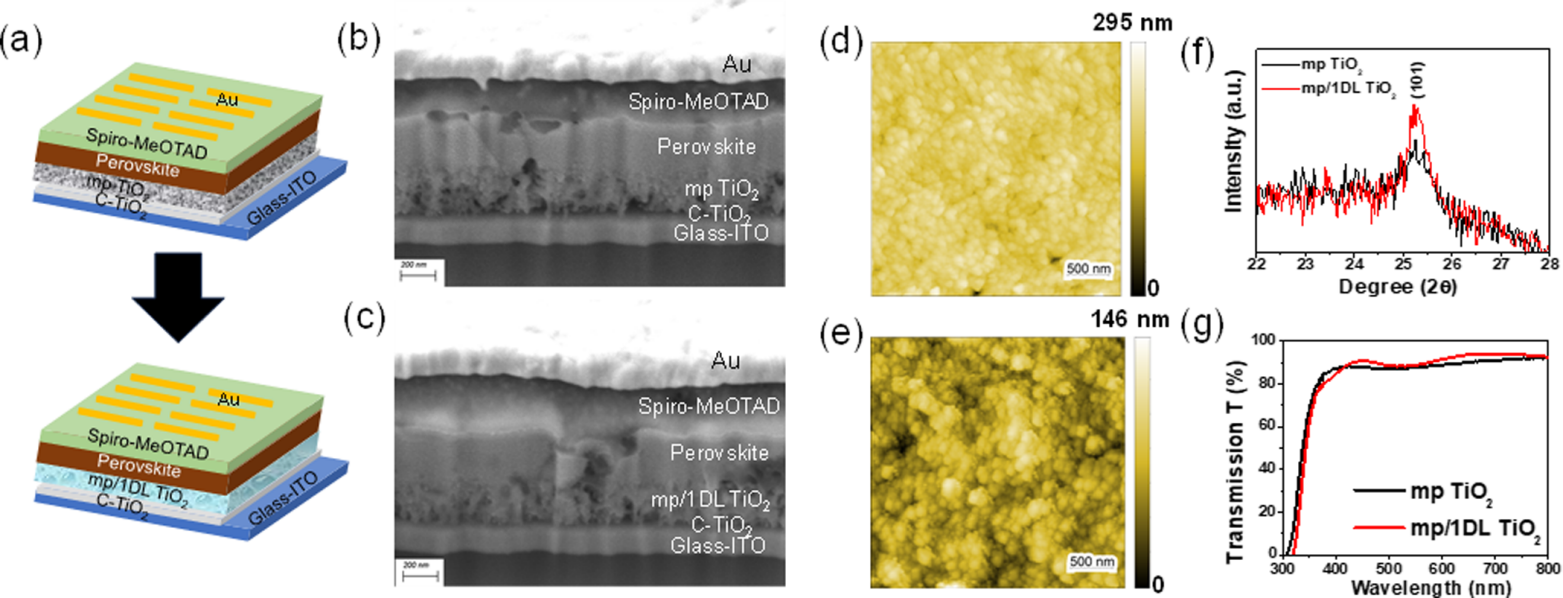
Structure and characterization of PSCs tested herein. (a) Schematic diagram of the device structure before (top) and after (bottom) introduction of 1DL as an interfacial layer. (b) Cross-sectional SEM image of PSCs before (top panel) 1DL layer deposition. (c) Cross-sectional SEM image of PSCs after the deposition of the 1DL layer on mp TiO_2_ (bottom panel). (d) AFM map of mp TiO_2_ and (e) AFM map of mp/1DL TiO_2_ layers on ITO. (f) XRD patterns of mp TiO_2_ (bottom black curve) and mp/1DL TiO_2_ layers (top red curve). (g) Transmission spectra of mp and mp/1DL TiO_2_ films (black and red curves, respectively).

A schematic of the cross-section of the layers in PSCs without and with a 1DL layer is shown in Figure 2a. Mesoporous PSCs were developed using a standard configuration described earlier.^40^ In some of the cells, before depositing the perovskite layer, a solution containing 1DL powders, composed of particles depicted in Figure 1e, was deposited onto the mp TiO_2_ using a two-step spin-coating technique. As discussed below, the deposited 1DL layer acts as a passivation layer between mp TiO_2_ and the perovskite (methylammonium lead iodide (CH_3_NH_3_PbI_3_), MAPbI_3_) film. Device fabrication details can be found in the Experimental Section below.

To demonstrate the successful deposition of the 1DL layer, we compared cross-sectional SEM images of PSCs without (Figure 2b) and with the 1DL (Figure 2c) layer. Analyzing these images, it is difficult to identify a distinct 1DL layer between the layers of mp TiO_2_ and MAPbI_3_ films. This situation arose due to the notably low concentrations of 1DL that were used. Therefore, to observe the impact of this deposition on mp TiO_2_, we employed atomic force microscopy (AFM) to examine the changes in the morphology induced by this effect. Figure 2d,e shows the topography maps for the mp TiO_2_ surface before and after 1DL deposition, respectively. The average roughness values for only mp TiO_2_ and mp/1DL are 189.9 ± 3.183 × 10^–06^ and 61.49 ± 6.571 × 10^–07^ nm, respectively (Figure S1). This indicates that after the 1DL layer deposition, the combined mp/1DL film’s roughness is significantly reduced, which is a crucial factor for better perovskite layer formation for high-performance PSCs.

Typical XRD patterns for the two ETLs are shown in Figure 2f. The diffractograms confirm the presence of anatase TiO_2_, as evidenced by the small peak corresponding to the (101) crystallographic plane for both ETLs. The higher peak intensity observed for mp/1DL TiO_2_ compared to mp TiO_2_ can be attributed to enhanced crystallinity.^41^Figure S2 presents the XRD spectra on a large scale for both ETLs, showing the absence of any additional peaks. Figure 2g compares the transmission spectra for both mp and mp/1DL TiO_2_ films. In both cases, the optical transmission in the visible range is high and wide. Also, Table S1 presents the electrical properties of mp TiO_2_ and mp/1DL TiO_2_ ETLs obtained from Hall measurements. The mp/1DL TiO_2_ ETL exhibits a higher carrier concentration, conductivity, and Hall mobility compared to mp TiO_2_, indicating superior electrical properties. The increased carrier concentration indicates a greater number of free electrons available for conduction, while the higher Hall mobility signifies that these carriers can move more freely under an applied electric field. These enhanced electrical properties contribute directly to more efficient charge transport and reduced recombination, leading to improved device performance.

**Figure 3:**
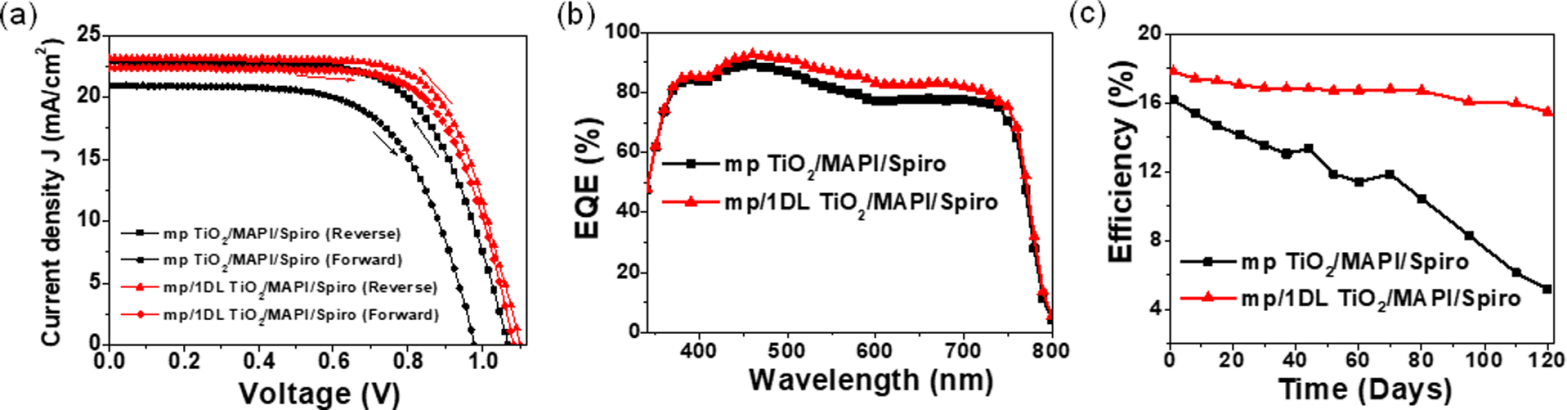
Comparison of the characteristics of PSCs having mp and mp/1DL TiO_2_ ETLs. (a) *J–V* curves during forward and reverse scans, (b) EQE spectra, and (c) efficiency stability plots.

The impact of the 1DL concentration on the performance of the PSCs was investigated by varying the concentrations of 1DLs in solution prior to their deposition. Figure S3 compares the current–density voltage (*J–V*) profiles for 7 different PSCs prepared using different 1DL concentration (in ethanol) solutions from 0 to 2.00 mg/mL. Table S2 summarizes the corresponding devices’ performance parameters, including *V*_OC_, *J*_SC_, fill factor (FF), and PCE. From these results, it is clear that the 0.10 mg/mL 1DL solution yielded the best performance and influenced the device performance significantly. Figure 3a compares the *J–V* curves for the optimal PSCs during forward and reverse scans. Table 1 summarizes the critical device performance parameters, viz. *J*_SC_, *V*_OC_, FF, and PCE. A control sample, viz. PSC without 1DL, resulted in a *V*_OC_ of 1.07 V, *J*_SC_ of 22.90 mA/cm^2^, and FF of 0.66, and PCE of 16.17% (under reverse scan). After 1DL layer (from the 0.10 mg/mL solution) deposition on mp TiO_2_, the PSC performance was considerably enhanced to 17.82% (under reverse scan). This enhancement stems from a significant increase in *V*_OC_ to 1.10 V and *J*_SC_ to 23.15 mA/cm^2^ compared with the pristine PSC. High-quality PSCs promote efficient charge collection, resulting in PSCs with less hysteresis. The reduction in hysteresis loss is probably attributable to a lower number of defects at the interface of combined ETL (mp/1DL TiO_2_) and perovskite than the control sample for well-developed surface coverage of the perovskite layer on 1DL, which is described in the next section of the manuscript.^40,42^

**Table 1:** Summary of *J*_SC_, *V*_OC_, FF, and % Efficiency of PSCs with mp Only and mp/1DL ETLs

ETL	*J*_SC_(mA/cm^2^)	*V*_OC_ (V)	FF	PCE (%)
mp TiO_2_				
(reverse)	22.90	1.07	0.66	16.17
(forward)	20.97	0.98	0.64	13.15
mp/1DL TiO_2_				
(reverse)	23.15	1.10	0.70	17.82
(forward)	22.43	1.08	0.69	16.71

Figure 3b,c compares the external quantum efficiency (EQE) spectra and efficiency stability plots for the corresponding devices, respectively. The EQE spectra for the mp/1DL TiO_2_-based PSC show a slightly higher intensity than the control device in the full wavelength range due to better light absorption.^43^ The *J*_SC_ values slightly increased through interface modification presumably due to better charge extraction, as indicated by the EQE spectra (Figure 3b). The integrated *J*_SC_ values derived from the EQE spectra for both devices are provided in Figure S4. These values corroborate that the mp/1DL TiO_2_-based PSC achieves a superior *J*_SC_ value compared to the control device, indicating a notable improvement in performance. The integrated *J*_SC_ value for the mp TiO_2_-based cell is 19.94 mA/cm^2^, while for the mp/1DL TiO_2_-based cell, it is 21.07 mA/cm^2^. The integrated *J*_SC_ value derived from the EQE spectra is slightly lower than the *J*_SC_ values measured under AM 1.5G one sun illumination. This slight discrepancy is typically observed because the EQE measurement uses a monochromatic light source and measures the response at discrete wavelengths, which may not perfectly replicate the solar spectrum under AM 1.5G conditions.

The use of 1DLs not only exhibited a remarkable enhancement in the PV performance but also resulted in considerable stability improvements. As shown in Figure 3c, the PCEs under ambient conditions were a strong function of the presence of the 1DL layer. After 120 days, the drop in efficiency of the 1DL-containing PSC (red curve in Figure 3c) was 13.1%; the corresponding value for the mp TiO_2_-based solar cell decreased by 67.9% (black curve in Figure 3c).

Figure S5 also depicts the operational stability graph for both PSCs under continuous 1 sun illumination for 500 s. The PCE of the PSCs containing 1DL TiO_2_-based NFs exhibited a decrease of only 7.68% after nearly 500 s of continuous illumination (red curve in Figure S5). In contrast, the PSCs based on mp TiO_2_ showed a significantly larger drop in PCE, with a reduction of 25.35% under the same conditions (black curve in Figure S5). These results clearly demonstrate that the 1DL TiO_2_-based NFs contribute to significantly enhanced operational stability of the PSCs under continuous illumination.

**Figure 4:**
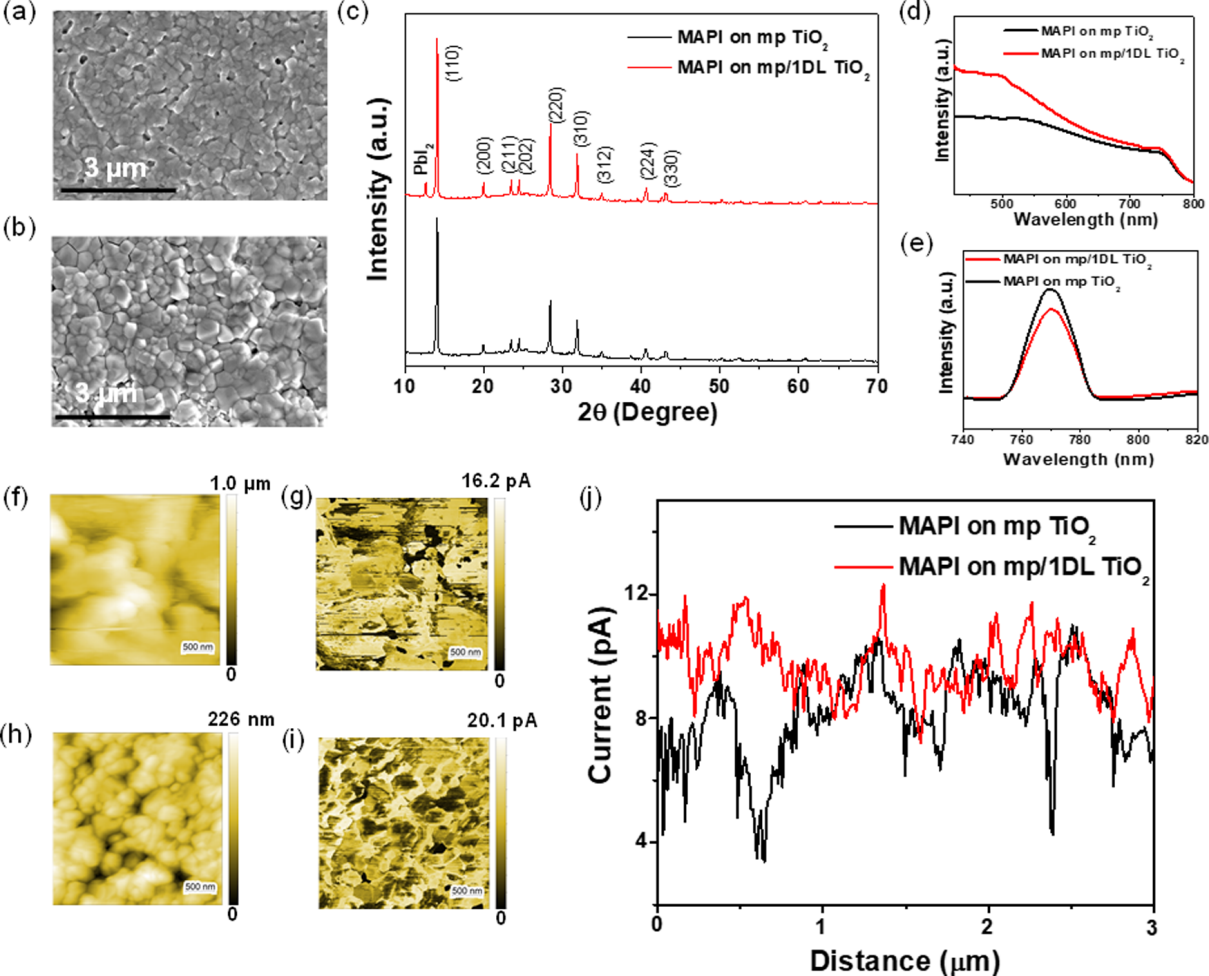
Characterization of the perovskite films (MAPbI_3_ is denoted as MAPI inside figure) deposited on mp TiO_2_ and mp/1DL ETLs: (a, b) FESEM micrographs, (c) XRD patterns, (d) UV/vis absorption, and (e) PL spectra. (f, h) AFM topography images and (g, i) corresponding pcAFM photocurrent images of the perovskite layers deposited on mp TiO_2_ and mp/1DL TiO_2_ ETL_S_, respectively. (j) Photocurrent line profiles across the perovskite layers.

The PV performance of PSCs can be affected by several parameters. For instance, the photocurrent generation is directly influenced by how well the active layer harvests light.^44^ Also, for high-performance PSCs, the most important parameter is the quality of the perovskite film used.^45,46^Figure 4a,b shows the SEM images of the top surfaces of the MAPbI_3_ films on mp TiO_2_ and mp/1DL ETLs, respectively. Both samples exhibit smooth and dense films at a macroscopic level that appear to be quite similar. However, when the grain size distributions are compared in Figure S6, it is clear that grains are, on average, larger for the 1DL-containing films than in their absence (compare red and black distributions in Figure S6). As importantly, the use of the 1DLs resulted in a reduction in the number of pinholes. The nucleation and growth of the perovskite film are affected by interface passivation, which can result in less pinhole-based surfaces.^47,48^ The black and red curves in Figure 4c show typical XRD patterns for the MAPbI_3_ films on mp and mp/1DL TiO_2_ ETLs, respectively. In both cases, the intense (110) peak around 2θ = 14.2° for the polycrystalline MAPbI_3_ films is clearly visible. Only one extra diffraction, the (001) peak for PbI_2_, appears around 12.5° (2θ), for the 1DL-based MAPbI_3_ films. The nucleation of a PbI_2_ secondary phase, due to interface modification, has been shown to improve the device performance.^49−51^ The formation of the PbI_2_ phase in our system is likely induced by the modified crystallization kinetics, resulting from the presence of the 1DL TiO_2_ layer at the interface. While the exact mechanisms remain to be explored, this finding underscores the significant impact of the interface on the structural and electronic properties of the perovskite layer. Figure 4d shows the absorption spectra of the perovskite films on mp (black curve in Figure 4d) and mp/1DL ETLs (red curve in Figure 4d), respectively. Below 700 nm, the latter absorbs more light. This indicates that the enhancement in the EQE spectrum for the 1DL-based PSCs can, in part, be attributed to a higher light absorption.

We also used steady-state photoluminescence (PL) to examine the recombination kinetics of the perovskite films formed on both ETLs. Figure 4e demonstrates the steady-state PL spectra of the perovskite films without (black in Figure 4e), and with (red in Figure 4e), 1DL TiO_2_-based ETLs. Under similar experimental conditions, both perovskite films exhibit a PL peak at about 770 nm. The PL intensity for the 1DL-containing film (red curve), however, is reduced compared to that of the control film (black curve). This lower PL intensity suggests that the 1DL-modified ETL improves charge carrier extraction. Efficient extraction reduces the level of recombination of electrons and holes at the interface, thereby supporting our claim of decreased charge recombination.

To get a deeper understanding of the enhanced photocurrent production within the perovskite layer, we used photoconductive atomic force microscopy (pcAFM) to map the photocurrent distribution at the nanoscale for the same perovskite layers on both types of ETLs. The topography (Figure 4f,h) and the corresponding local photocurrent images (Figure 4g,i) of the perovskite layers on mp TiO_2_ and mp/1DL TiO_2_-based ETLs are shown, respectively. The corresponding photocurrent spectra in Figure 4j show that the generation of the current across the 1DL TiO_2_-based perovskite layer is higher than that for the control sample. This increased photocurrent corresponds to more efficient charge collection and transport, indicative of fewer recombination events. Based on these results, it is again reasonable to conclude that the 1DL-containing devices show enhanced electron densities and higher conductivities, which are consistent with the improved performances of the PSCs.

**Figure 5:**
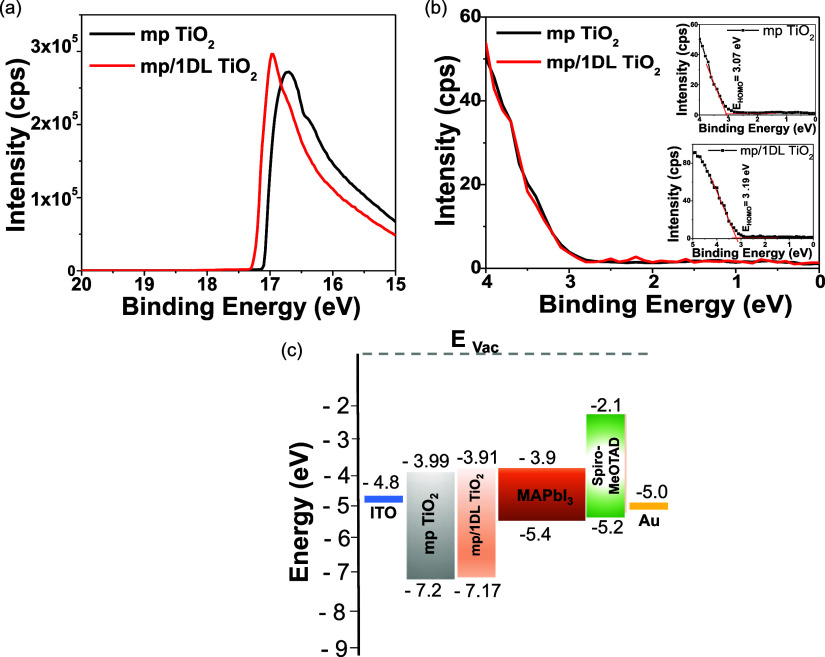
Characterization of electronic structures of various ETLs: (a) UPS spectra for mp TiO_2_ (black line) and mp/1DL TiO_2_ (red line) ETLs. (b) Valence band edge region for ETLs. Top and bottom insets show *E*(HOMO) values for mp TiO_2_ and mp/1DL TiO_2_ ETLs, respectively. (c) Energy diagrams, vs vacuum, for each layer in the PSC device with energy levels in eV.

The higher efficiencies of the PSCs after interface engineering can be explained by the fact that their band positions are slightly better matches than the control. To determine the energy levels of the ETLs, with and without 1DL, we carried out UV–vis absorption and UV photoelectron spectroscopy (UPS) experiments. Figure 5a compares the UPS spectra of the mp TiO_2_ without and with 1DL. Table S3 summarizes the photoemission parameters obtained from the UPS experiments. The values of the highest occupied molecular orbital (*E*_HOMO_) can be determined from the UPS spectra shown in Figure 5b. The calculated *E*_VB_ values are −7.20 and −7.17 eV for mp and mp/1DL TiO_2_, respectively. From the UV–vis absorption spectra (Figure S7b,d), the band gaps for the sample with only mp TiO_2_ and mp/1DL samples were estimated from Tauc plots to be 3.21 and 3.26 eV, respectively.

With this and other information, the energy level diagrams of the layers in the device can be plotted (Figure 5c). The alignment of these levels facilitates the separation of the charge carriers across the interfaces and their transport through the layers. In the case of 1DL TiO_2_-treated ETL, there is a slightly better alignment of the energy levels at the ETL/perovskite, leading to possibly easier electron extraction and enhanced performance of corresponding PSCs.^18,40,52^ The enhancement, however, is small, and further work, outside of the scope of this paper, is needed to focus/confirm this aspect.

To further validate the effectiveness of our fabrication strategy, *J–V* performances of the PSC devices with mp TiO_2_ and mp/1DL ETLs were systematically evaluated, and the histograms of *V*_OC_, *J*_SC_, FF, and PCE for other devices are shown in Figure S8. The improvement in the *V*_OC_ and *J*_SC_ values, together with the slight increase in FF, is credited with the enhancement in the PCE. The better matching in energy levels between the ETL and perovskite leads to enhanced electron transfer through the implementation of interface engineering and demonstrates an easy and effective way to improve the performance of the PSCs.

## Conclusions

Herein, we utilized new 1DL TiO_2_-based NFs to modify the interface between the mp TiO_2_ and perovskite layers in PSCs. The results prove this new material to be a promising material for enhancing the efficiency, stability, and hysteresis-free performance of PSCs. The charge transfer efficiency at the interface of the ETL and perovskite was enhanced through the interface modification. We also found that 1DL induced the formation of perovskite films with larger grain sizes, good crystallinity, and presumably lower trap densities.

The passivation effects significantly boosted the PCE from 16.17 to 17.82% with the enhancement of *V*_OC_ from 1.07 to 1.10 V, compared to only mp TiO_2_-based solar cells. Moreover, PSCs with mp/1DL ETLs retained ∼87% of their initial performance after 120 days in air without encapsulation, indicating that the solar cell stabilities were also significantly improved after the interface modification.

In summary, the use of 1DL TiO_2_, a new and also valuable material for advancing the development of efficient and stable PSCs, could perhaps result in the creation of a new pathway for the development of commercially viable and stable devices. The fact that we currently readily, simply, and routinely produce these materials, at near ambient conditions, in 100 g batches in the lab today, starting with inexpensive precursors, bodes well for their commercial adoption in PSCs. More work, however, is needed and indicated. Here, we started with TiB_2_ powder, reacted it at 80 °C for 5 days with TMAH, and washed it to produce the mesostructures shown Figure 1e. Among the unknowns at this stage is what role the 1DL’s processing conditions, morphologies, and choices of precursor chemistry play in this application. In other words, if we were to begin with other Ti-containing compounds reacted with quats other than TMAH, with times and temperatures different than those used here and ending with quasi-2D flakes instead of mesostructures shown Figure 1e, would this alteration impact device performances? Arguably, the answer must be yes since it is reasonable to conclude that further research can enhance the device’s performance. What we present here is the first generation.

## Experimental Section

### Materials

Glass/indium tin oxide (ITO)-coated substrates (commercial) were used for device fabrication. Titanium isopropoxide (Ti(OC_3_H_7_)_4_, Sigma-Aldrich, 99.999%), ethanol (CH_3_CH_2_OH, Sigma-Aldrich), and acetylacetone (C_5_H_8_O_2_, Fisher Scientific, ≥99%) were used to deposit compact TiO_2_ layers, and titania paste (791547-20 G, Sigma-Aldrich) was utilized as a precursor for the mp TiO_2_ layer.

Titanium diboride (TiB_2_, −325, Thermo Scientific, PA) powders, tetramethylammonium hydroxide aqueous solution (TMAH, 25 wt % in DI water, 99.9999%, Alfa Aesar, PA), ethanol (Decon Lab Inc., 200 proof, PA), DI water (Millipore, 18.2 MΩ, TOC < 3 ppb), and lithium chloride (LiCl, Anhydrous, 99%, 20 mesh, Alfa Aesar, PA) were used to prepare 1DL powders.

To make the MAPbI_3_ perovskite solution, lead(II) iodide (PbI_2_, Sigma-Aldrich, 99%), methylammonium iodide (MAI/CH_3_NH_3_I, Sigma-Aldrich, 98%), dimethylformamide (DMF), and DMSO from Sigma-Aldrich were used. To produce the HTL, a mixture of chlorobenzene (C_6_H_5_Cl, Sigma-Aldrich, 99.8%) solution with Spiro-MeOTAD (Sigma-Aldrich, 99%), 4-*tert*-butylpyridine (96%, Sigma-Aldrich), and bis(trifluoromethane)sulfonimide lithium salt (Li-TFSI, Sigma-Aldrich, ≥99.0%) was used.

### Device Fabrication

The glass/ITO substrates were sequentially cleaned by using a soap solution, distilled water, acetone, and isopropanol after etching. Compressed air was used to dry the substrates. Before the compact layer was deposited, the surfaces were treated for 15 min with UV–ozone at 60 °C. The TiO_2_ compact layer was deposited by spin-coating a solution of titanium isopropoxide in ethanol and acetylacetone at 4000 rpm for 35 s. The samples were then dried at 150 °C for 15 min and then annealed for 30 min at 500 °C. The mp TiO_2_ layer was spin-coated (at 4000 rpm for 20 s) starting with a solution of 0.31 g of TiO_2_ paste in 2 mL of ethanol. After that, the samples were heated in different heating steps (325 °C for 30 min, 450 °C for 15 min) and then finally annealed at 500 °C for 30 min.

### 1DL TiO_2_ Preparation

1DL TiO_2_ powders were prepared by mixing 20 g of TiB_2_ commercial powders with 200 mL of 25 wt % TMAH in a plastic bottle. Two fine needles were inserted in the bottle to avoid pressure buildup during the reaction. The mixture was shaken at 80 °C for 5 days using a temperature-controlled shaking incubator (Labnet 211DS, 49L, 120 V, NJ) under ambient pressure. After the reaction, the resulting slurry was combined with ∼200 mL of ethanol in a beaker (1 L in size) and stirred for 1–2 h using an overhead mixer, before allowing the mixture to settle down. The supernatant (composed mostly of excess TMAH salt and other soluble reaction products) was then discarded. This washing process was repeated multiple times until a pH ≈ 7 was reached.

At this stage, the resulting powders are 1DL TiO_2_-based porous hierarchical particles (Figure 1e) with TMA cations in the interfilamentous gallery. To exchange the TMA^+^ intercalants with Li^+^ ones, the powders were transferred straight from ethanol to a 5 M LiCl aqueous solution and stirred for 6 h under ambient conditions. This step was carried out 3 subsequent times to ensure full cation exchange. The powders were then rinsed with DI water for 4 cycles to remove any unreacted LiCl salts or reaction byproducts. Lastly, the 1DL particles were dried at 50 °C in the open air overnight.

The dry powders (i.e., 1DL TiO_2_-based porous hierarchical particles) were then mixed with ethanol to form suspensions with concentrations ranging from 0.05 to 2 mg/mL. All samples were sonicated for 10 min to ensure homogeneous dispersion. The suspensions were then deposited on the mp TiO_2_ layer using a two-step spin-coating technique (600 rpm for 20 s and 1500 rpm for 10 s).

For the perovskite films, PbI_2_ (215 g) was dissolved in a mixture of DMF and DMSO at 70 °C, followed by the addition of MAI (622 g) and 24 h of constant stirring. This perovskite solution was spin-coated on mp/1DL TiO_2_ layers using a two-step spinning process (at 1000 rpm for 10 s and 5000 rpm for 30 s) and then heated at 100 °C for 15 min in air. To make the Spiro-MeOTAD solution, 54.2 mg of Spiro-MeOTAD was mixed with 0.75 mL of chlorobenzene. As components, the solution contained the LiTFSI stock solution (520 mg of LiTFSI in 1 mL of acetonitrile) and 21.37 μL of 4-tBP. Finally, a 100 nm Au electrode was formed using thermal evaporation and the shadow mask method (Vinci Technologies). The active device area was 0.1 cm^2^.

### Characterization Details

#### Scanning Electron Microscopy

A scanning electron microscope (Carl Zeiss AURIGA Cross Beam workstation) was used to image the morphology of mp and mp/1DL TiO_2_ and perovskite layers. Using a conventional Everhart–Thornley secondary electron detector and 30 kV Ga^+^ ions at 20 pA, we captured cross-sectional images of the solar cells.

Another scanning electron microscope (Zeiss Supra 50 VP, Carl Zeiss SMT AG, Oberkochen, Germany) was used to obtain micrographs of the 1DL powders. The SEM settings were set to an in-lens detector, a 30 mm aperture, and an accelerating voltage of 3–5 kV.

#### X-ray Diffraction

XRD patterns of the 1DL TiO_2_ powders were obtained by using a diffractometer (Rigaku MiniFlex, Tokyo, Japan) operated with Cu K_α_ radiation (40 kV and 15 mA). The powders were scanned in the 2–65° 2θ range with a step size of 0.02° and a dwell time of 1 s.

#### Atomic Force Microscopy

All AFM measurements were performed using an Asylum Research MFP-3D system (Oxford Instruments, UK). pcAFM measurements were taken in air by using conductive PtIr-coated silicon AFM tips (Nanoworld CONTPt, resonance frequency = 13 kHz, spring constant = 0.2 N/m) and an ORCA-2 current detector holder. A Fiber-Lite MI-150R light source was used to light the samples.

#### Transmission Electron Microscopy and Selected Electron Diffraction

TEM (JEOL, JEM2100F field-emission TEM) was used to image the samples. SAD patterns of select 1DL powders were also collected. TEM was operated at 200 keV and had an image resolution of 0.2 nm. Images and diffraction patterns were collected on a Gatan USC1000 CCD camera.

#### X-ray Photoelectron and Ultraviolet Photoelectron Spectroscopy

XPS analysis of select films was performed with a spectrometer (Axis Supra by Kratos Analytical, Shimadzu Group Company, Japan) using monochromatic Al K radiation (X-ray power = 300 W and pass energy = 40 eV).

The UPS analysis was carried out in an ultrahigh vacuum chamber using a helium, He, discharge lamp calibrated for He I emission (21.22 eV). To limit the intensity reaching the detector, a 55 μm diameter aperture was utilized. The pass energy was set to 5 eV, and the step size was 0.025 eV.

### Optical Spectra Measurements

Absorption and transmission spectra of the samples were measured using a UV–vis–NIR spectrophotometer (Agilent Cary series).

PL spectra were acquired by using a high-resolution spectrometer (Horiba Jobin Yvon, Model: iHR 320) and a photomultiplier tube.

#### Electrical Characterizations

Using a workstation (Sciencetech SS1.6 kW-A-2Q system with Keithley source meter: Model 2400) under a LED Solar Simulator VeraSol LSH-7520 (USA) with AM1.5 G (1 sun conditions, 100 mW cm^–2^), current density–voltage (*J–V*) studies were obtained. A xenon lamp (Newport, USA) paired with a monochromator (Newport, USA) was utilized to measure the EQE.

## Supporting Information Available

3D AFM topography images of mp TiO_2_ and mp/1DL TiO_2_ on ITO and corresponding roughness plot; XRD spectra for mp TiO_2_ and mp/1DL TiO_2_ ETLs; electrical properties of mp TiO_2_ and mp/1DL TiO_2_ ETLs obtained from Hall measurements (in table); *J–V* characteristics of the solar cells for different concentrations of 1DL TiO_2_; *J–V* parameters of the solar cells for different concentrations of 1DL TiO_2_ (in table); EQE spectra and spectrally integrated current density of the best-performing PSCs with mp TiO_2_ and mp/L TiO_2_ ETLs; operational stability for both PSCs under continuous 1 sun illumination for 500 s; grain size distribution for two different MAPbI_3_ layers deposited on mp TiO_2_ and mp/1DL TiO_2_-based ETLs; work function (φ), E (HOMO), and E (VB) values (eV) for both mp TiO_2_ and mp/1DL TiO_2_-based ETLs (in table); UPS spectra and band gap derived from the UV–vis measurements for mp TiO_2_ and mp/1DL TiO_2_ ETLs; and box diagrams of the measured *J–V* parameters of the solar cell batches with mp TiO_2_ and mp/1DL TiO_2_ ETLs. It contains the supporting figures (PDF)
