# Supplementary material for: Interfacial Engineering with One-Dimensional Lepidocrocite TiO2-Based Nanofilaments for High-Performance Perovskite Solar Cells
Source: ACS Omega. 2024 Dec 13;9(51):50820–9. doi: 10.1021/acsomega.4c09516 (PMC11683603; doi:10.1021/acsomega.4c09516)
Supplement: Supplementary file 1 — ao4c09516_si_001.pdf [file ao4c09516_si_001.pdf]

Supporting Information

Interfacial Engineering with One-dimensional Lepidocrocite TiO_2-based Nanofilaments for High Performance Perovskite Solar Cells

Shrabani Panigrahi*,[1] Hussein O. Badr,[2] Jonas Deuermeier,[1] Santanu Jana,[1] Elvira Fortunato,[1] Rodrigo Martins,[1] Michel W. Barsoum*[2]

[1]i3N/CENIMAT, Department of Materials Science, NOVA School of Science and Technology, and CEMOP/UNINOVA, Campus de Caparica, 2829-516 Caparica, Portugal.

[2]Department of Material Science and Engineering, Drexel University, Philadelphia, PA, USA

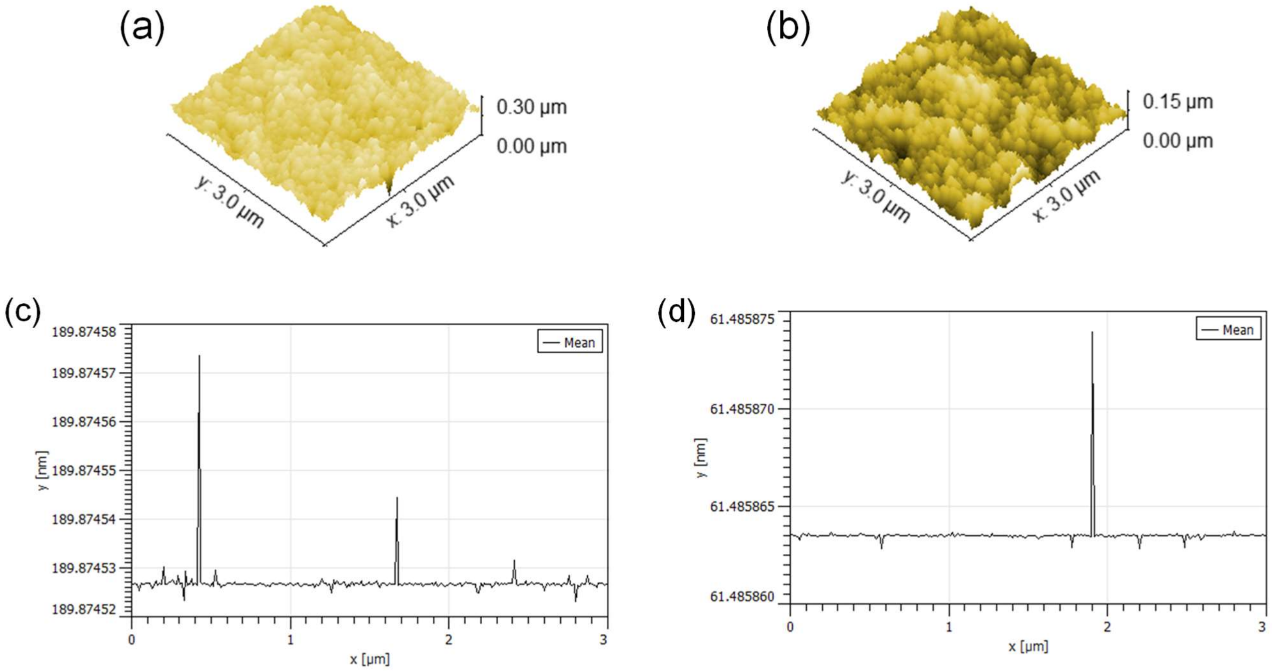

Figure S1: (a) and (b) 3D AFM topography images of mp TiO_2 and mp/1DL TiO_2 on ITO. (c) and (d) Corresponding roughness plot for two ETLs.

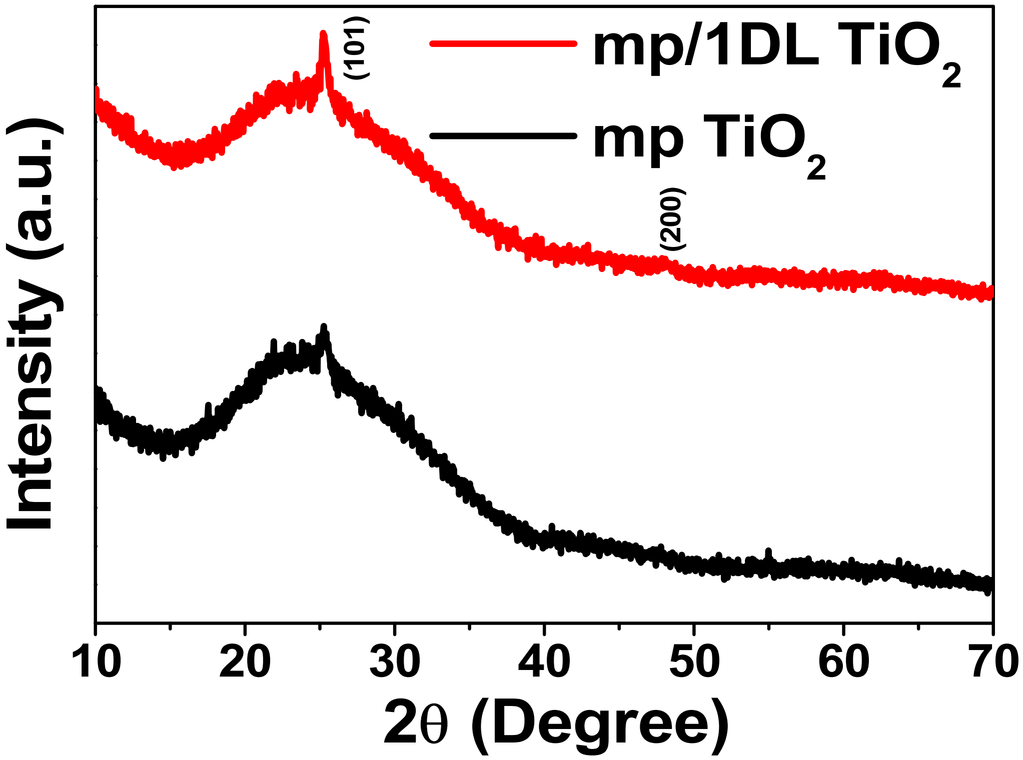

Figure S2: XRD spectra for mp TiO_2 and mp/1DL TiO_2 ETLs.

Table S1: Electrical properties of mp TiO_2 and mp/1DL TiO_2 ETLs obtained from Hall measurements.

Sample	Carrier Concentration (cm^{-3})	Conductivity (S/cm)	Hall Mobility ($Cm^3\ V^{-1}s^{-1}$)	Resistivity (Ω·cm)
mp TiO_2	$6.83 x 10^{19}$	4.17×10^4	40.50	2.40×10^{-5}
mp/1DL TiO_2	$3.18 x 10^{20}$	2.83×10^5	55.70	3.53×10^{-6}

The higher carrier concentration, conductivity, and Hall mobility observed in the mp/1DL TiO_2 ETL compared to mp TiO_2 indicate superior electrical properties crucial for electronic and optoelectronic applications.

The enhanced carrier concentration suggests a greater density of charge carriers available for conduction within the material. This attribute is directly linked to the improved conductivity of mp/1DL TiO_2, enabling more efficient transport of electrons through the layer.

Moreover, the higher Hall mobility signifies that charge carriers in mp/1DL TiO_2 can move more freely under an applied electric field, which is beneficial for achieving faster response times and improved device performance.

These superior electrical characteristics of mp/1DL TiO_2 ETL not only enhance the overall conductivity of devices but also contribute to their efficiency and operational stability in various technological applications.

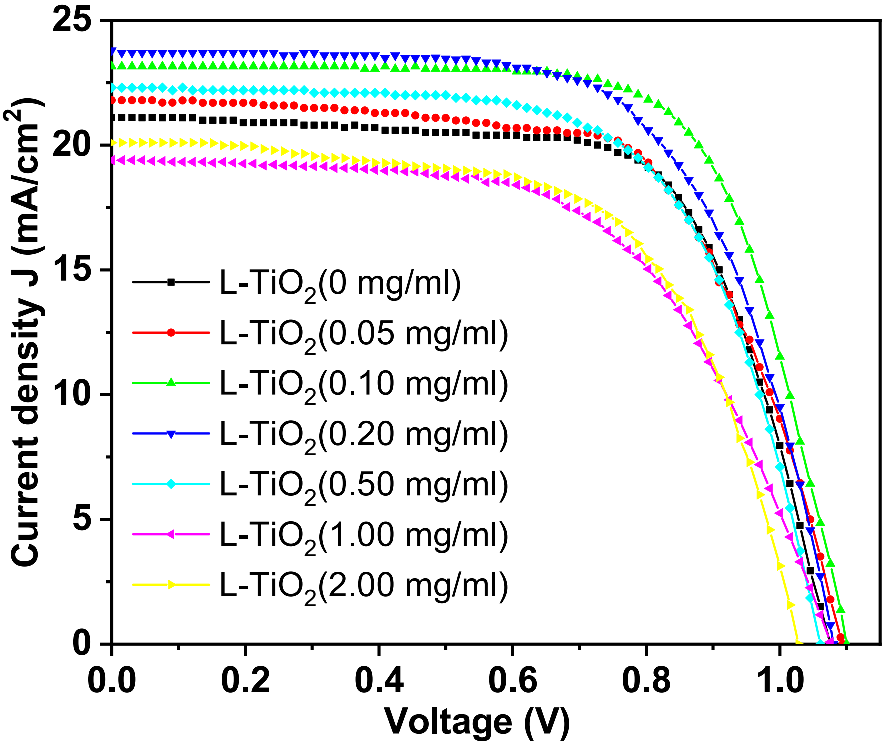

Figure S3: (a) *J-V* characteristics of the solar cells for different concentration of 1DL TiO_2.

We have produced others concentration of 1DL TiO_2 solution and developed different 1DL TiO_2 based solar cells. We also observed their electrical characteristics to show the best concentration of 1DL TiO_2 based solar cell. When the amount of 1DL TiO_2 is increased upto 0.10 mg/ml, V_{OC} increases due to perfect band alignment with perovskite, then again decreases when the amount is more increased.

Table S2: *J-V* parameters of the solar cells for different concentration of 1DL TiO_2.

ETL	J_{SC} (mA/cm^2)	V_{OC} (V)	FF	PCE (%)
mp TiO_2 (1DL TiO_2= 0 mg/ml)	21.10	1.08	0.68	15.49
mp/1D L TiO_2 for 1DL TiO_2 0.05 mg/ml	21.80	1.09	0.66	15.68
**mp/1DL TiO_2 for 1DL TiO_2 0.10 mg/ml**	**23.15**	**1.10**	**0.70**	**17.82**
mp/1DL TiO_2 for 1DL TiO_2 0.20 mg/ml	23.80	1.08	0.65	16.70
mp/1DL TiO_2 for 1DL TiO_2 0.50 mg/ml	22.30	1.06	0.65	15.36
mp/1DL TiO_2 for 1DL TiO_2 1.00 mg/ml	19.39	1.07	0.59	12.24
mp/1DL TiO_2 for 1DL TiO_2 2.00 mg/ml	20.10	1.03	0.62	12.83

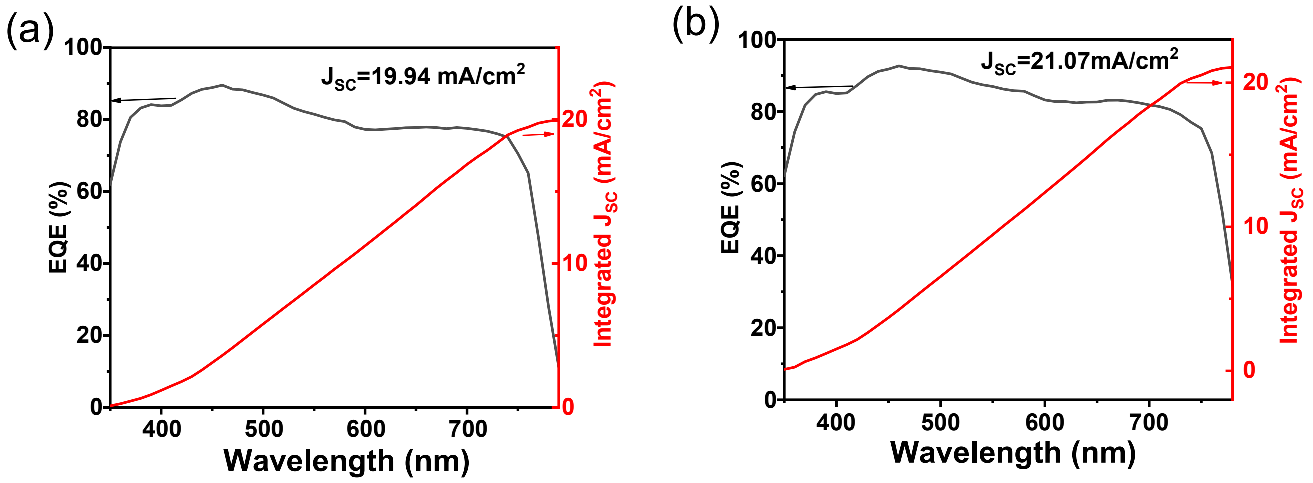

Figure S4. EQE spectra and spectrally integrated current density of the best-performing PSCs with (a) mp TiO_2 and (b) mp/L TiO_2 ETLs, respectively.

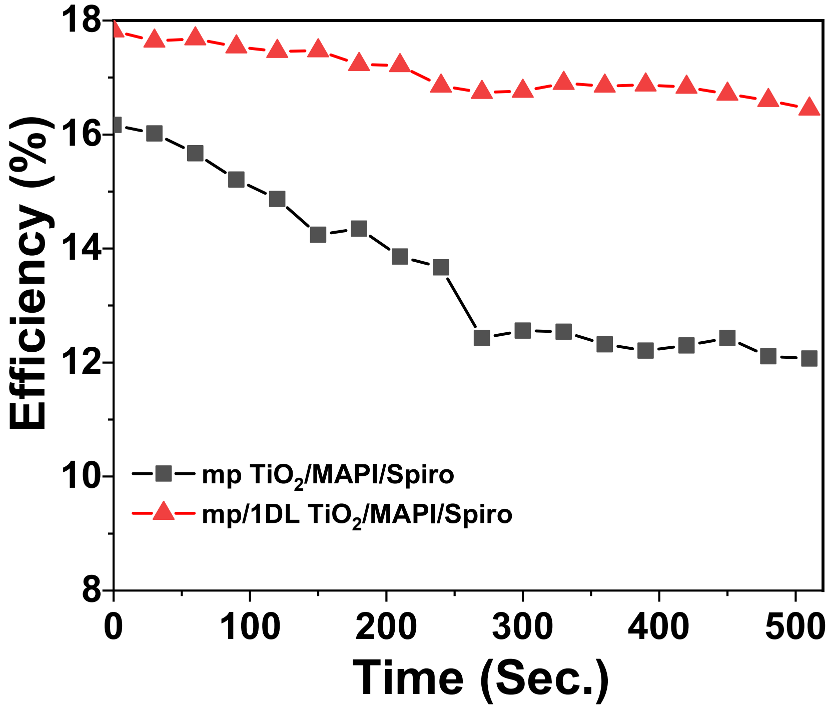

Figure S5: Operational stability for both PSCs under continuous 1-sun illumination for 500 S.

The power conversion efficiency (PCE) of the PSCs containing 1DL TiO_2-based nanofilaments exhibited a decrease of only 7.68 % after nearly 500 seconds of continuous illumination (red curve in Figure S5). In contrast, the PSCs based on mp TiO_2 showed a significantly larger drop in PCE, with a reduction of 25.35 % under the same conditions (black curve in Figure S5). These results clearly demonstrate that the 1DL TiO_2-based nanofilaments contribute to significantly enhanced operational stability of the PSCs under continuous illumination. The marked improvement can be attributed to the improved interface engineering. The 1DL nanofilaments provide better passivation of the perovskite layer, reducing trap states and minimizing nonradiative recombination.

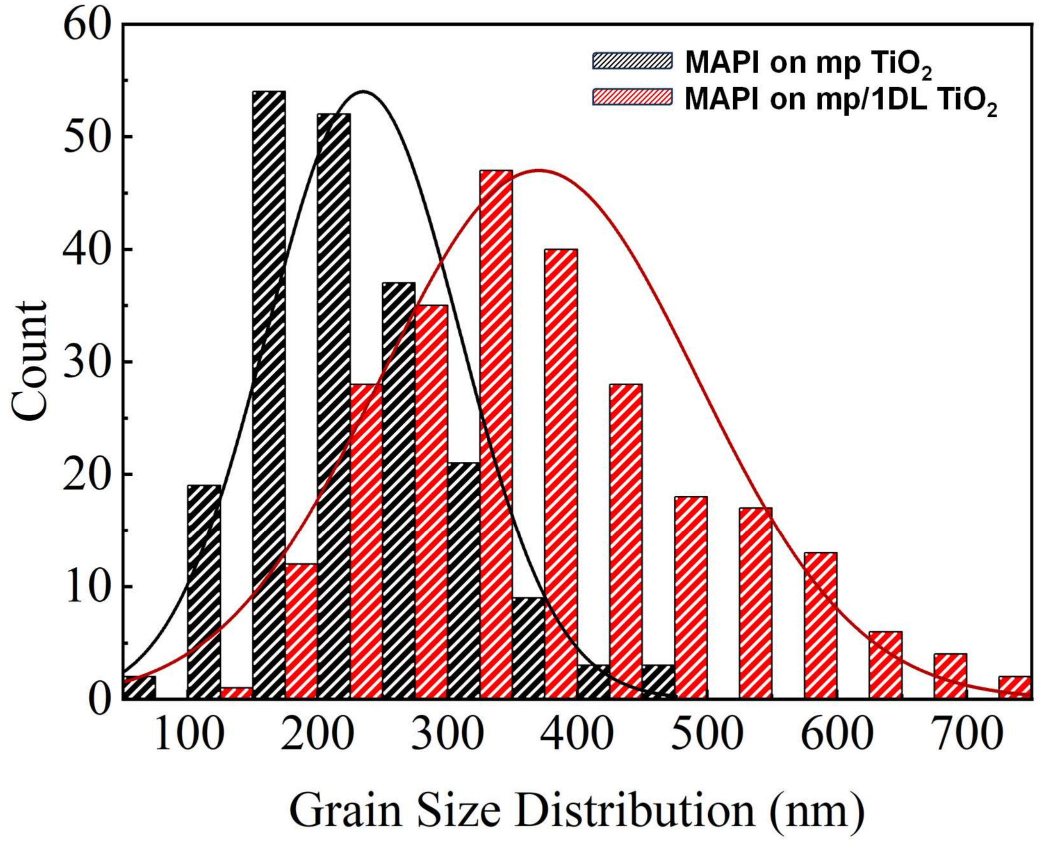

Figure S6: Grain size distribution for two different $MAPbI_3$ (denoted as MAPI inside figure) layers deposited on mp TiO_2 and mp/1DL TiO_2 based ETLs (black and red columns, respectively). Grain size measurements were carried out using ImageJ from corresponding SEM micrographs presented in Figs. 4 (a) and (b) (main manuscript), respectively.

Table S3: Work function (φ), E (HOMO), and E (VB) values - in eV - for both mp TiO_2 and mp/1DL TiO_2-based ETLs. All values were calculated from the UPS spectra shown in Figure 5a and b.

ETLs	Work Function (ϕ) (eV)	E_{HOMO} (eV)	E_{VB} (eV)
**mp TiO_2**	**4.13**	**3.07**	**7.20**
**mp/1DL TiO_2**	**3.98**	**3.19**	**7.17**

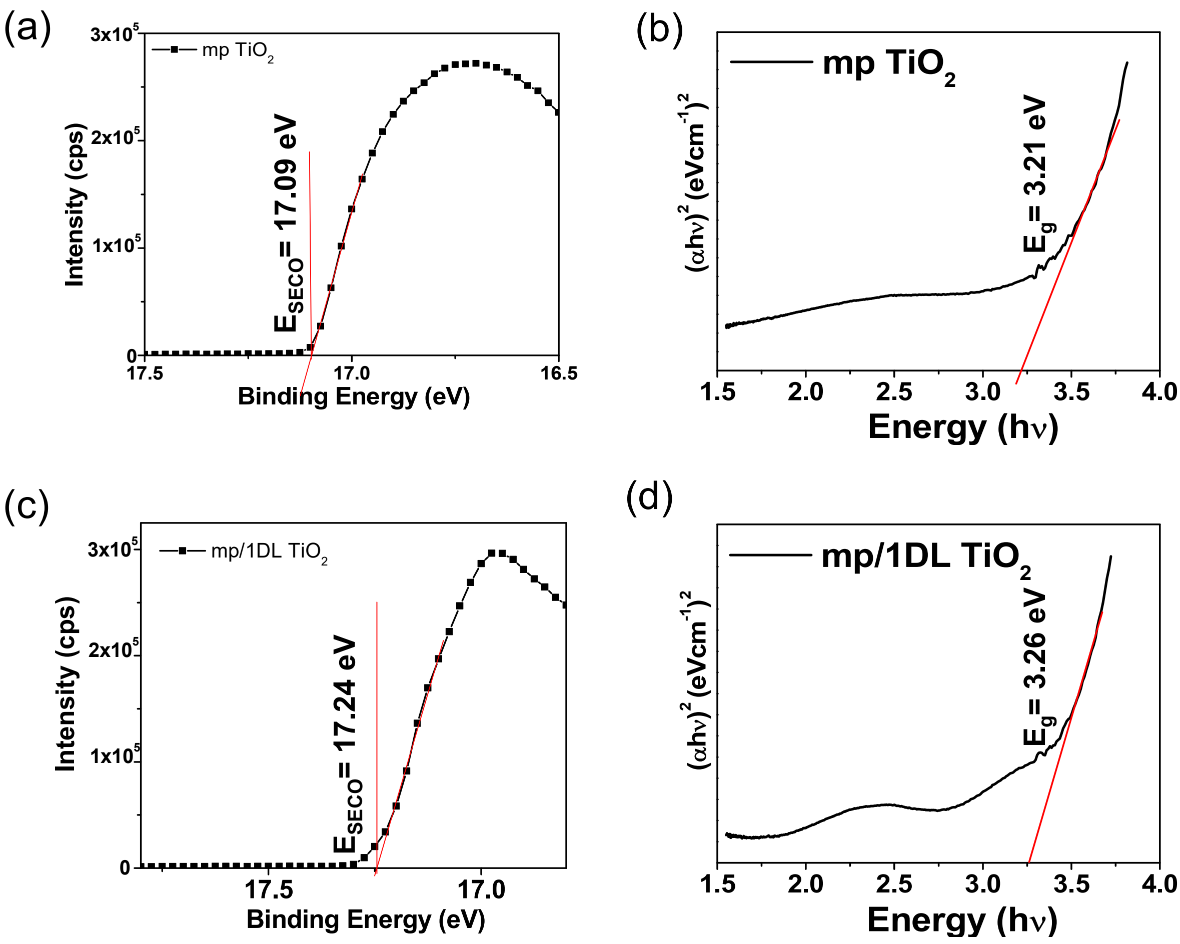

Figure S7: (a), (c) UPS spectra showing the cut-off region (E_{SECO} value) and (b), (d) bandgap derived from the UV–vis measurements for mp TiO_2 and mp/1DL TiO_2 ETLs, respectively.

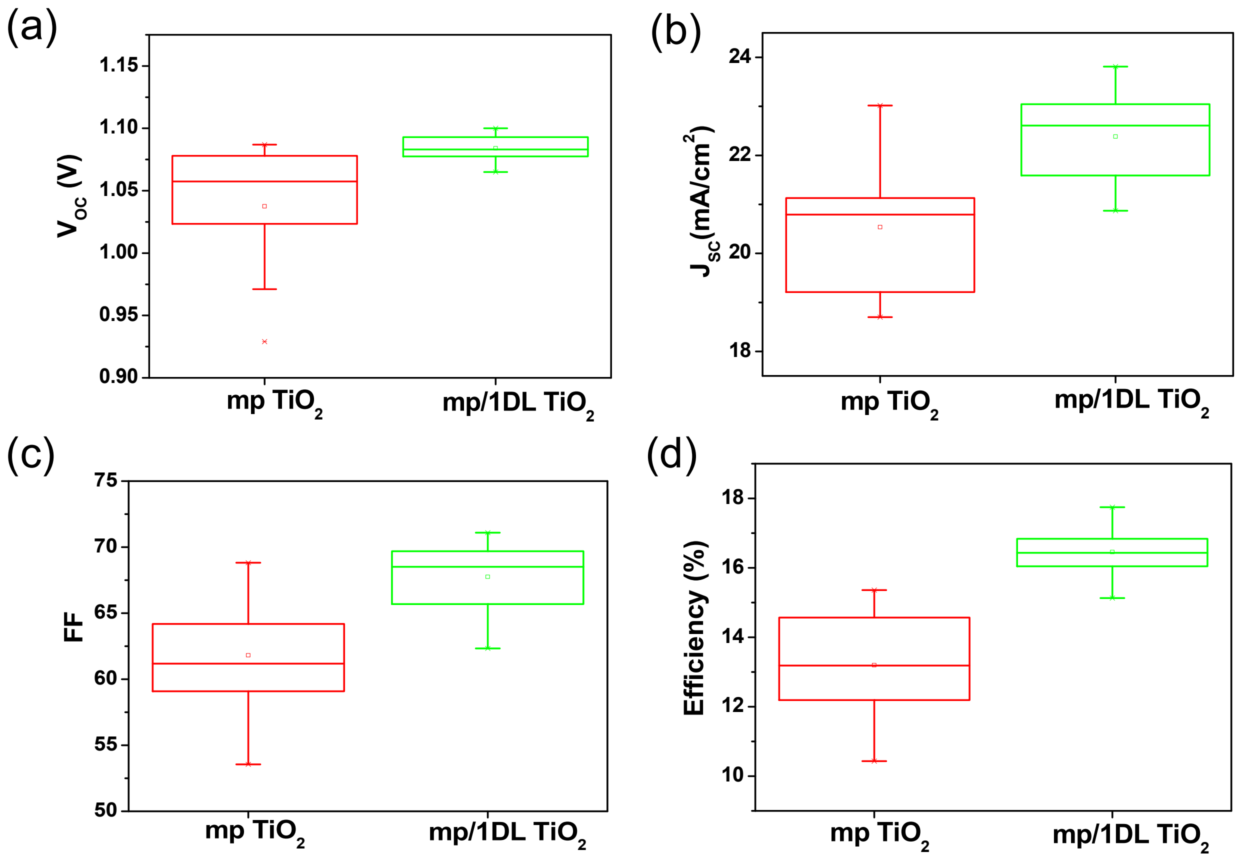

Figure S8: Box diagrams of the measured *J-V* parameters (a) V_{OC}, (b) J_{SC}, (c) FF, and (d) Efficiency of the solar cell batches with mp TiO_2 and mp/1DL TiO_2 ETLs, respectively.

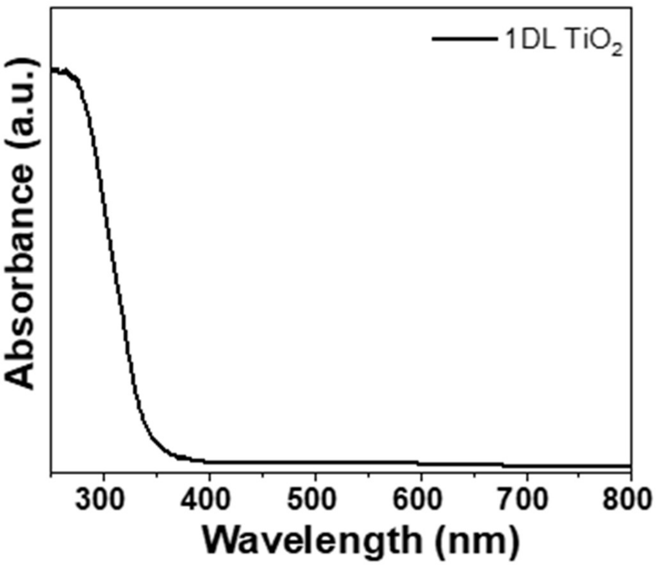

Figure S9: UV-Visible absorption spectrum of 1DL TiO_2.

We performed UV-vis absorption spectroscopy on the isolated 1DL TiO_2 NFs to assess their optical absorption characteristics. Based on known properties of TiO_2 nanostructures, we expect the absorption edge to be in the UV region due to TiO_2's wide band gap, similar to the values discussed in the manuscript (~3.21–3.26 eV). However, the presence of 1DL might slightly shift the absorption edge, reflecting changes in band structure and confirming its role in electron transport enhancement.

The absorption spectrum of 1DL TiO_2 will thus help demonstrate how the nanofilaments interact with light and contribute to the overall improved performance of the devices. We will include this data either in the main manuscript or supplementary materials.
